# In tribute to, in honor of, and with respectful memories of Professor William C. Shoemaker

**DOI:** 10.1186/s13054-016-1316-3

**Published:** 2016-07-22

**Authors:** Bart Chernow

**Affiliations:** Present address:, 4240 Galt Ocean Drive, Suite 2404, Fort Lauderdale, Florida 33308 USA

Accomplishment, recognition, innovation, discovery, respect, successes, titles, achievement, emulation, mentorship, growth, and completion of one’s objectives…coupled with impediments, peer resistance, frustration, uncertainty, disappointment, and occasional failures. These words represent the positive and occasionally negative experiences of many (if not most) physician-scientists who are dedicated to the tripartite mission of providing clinical care, discovery, and education within the broad field of medicine. In the case of Dr. William Shoemaker, the evidence clearly supports the statement that he accomplished much and experienced extraordinary achievements:

1. In his work as a scientist (with a number of highly talented co-workers) dedicated to exploring the hemodynamic responses to various forms of trauma and critical illness.
2. In his successful efforts to create (with some gifted colleagues) a specialty of multi-disciplinary critical care and an organization (the Society of Critical Care Medicine (SCCM)) to serve as a platform for the dissemination of clinical, research, educational, and organizational information which would facilitate growth of the field and provide numerous benefits to members and the patients they serve.
3. In service to the SCCM as its third President, as a frequent invited lecturer, and, of course, as the founding Editor-In-Chief of the SCCM journal, *Critical Care Medicine*.
4. In his mentorship (and encouragement) to many others…always with respect and humility.

Dr. Shoemaker was a prolific clinical investigator. He was the author or co-author of almost 700 scientific publications and 55 books or book chapters [1], including his role as lead editor (co-edited with several leading authorities) of the *Textbook of Critical Care* (an incredible amount of work—about 2200 pages and 200 chapters). However, the most noteworthy aspect of his career in clinical research was his focus on the physiologic monitoring of critically ill and traumatically injured patients. Specifically, he and his colleagues were dedicated to evaluating non-invasive hemodynamic monitoring systems as the best continuous way to identify diminished tissue perfusion and consequently impaired oxygen delivery. Dr. Shoemaker and his co-workers were not only focused on describing the hemodynamic and oxygen transport responses to various types of illness and injury but also analyzed the therapeutic implications of their observations. Dr. Shoemaker and his team recognized that early non-invasive hemodynamic monitoring might have important therapeutic and prognostic capabilities. Their work included studies of numerous patients suffering from conditions ranging from head injury to peripheral artery trauma [2–6]. They even used mathematical programs to attempt to facilitate prognostic considerations and therapeutic decision-making [7, 8]. Will was erudite is his approach to discovery and what evolved from this iconic figure, over decades of work, was a platform of findings in hemodynamics and oxygen delivery for others to build upon. For physician-scientists in critical care, this is a person worthy of emulation.

Clearly, the achievements far outweighed the occasional frustrations.

When Professor Vincent invited me to write an obituary about Professor Shoemaker, I was touched but also challenged. I had served as Associate Editor under Will and was honored to be chosen as his successor as Editor-In-Chief of *Critical Care Medicine*. Over time, I learned much from this gentle giant of critical care. He helped to teach me about hard work and dedication. His comments in the hallways of a convention center were often as meaningful as a scientific discussion in a lecture room. I emulated him in a number of ways; however, I rarely had the opportunity to say “thank you”. He worked hard and long hours. I cannot remember a time when his hands were free of manuscripts and/or data from a study he was undertaking. When Dr. Shoemaker stepped down as editor of the journal, Dr. Robert H. Demling honored Dr. Shoemaker in a wonderful editorial. In that piece, he described Dr. Shoemaker as “Will the Scientist”, “Will the Organizer”, “Will the Educator” and “Will the Editor” [9]. Will not only made the signal contribution of being one of the founders of a multi-disciplinary field, a multi-disciplinary society, and a multi-disciplinary journal but was also a multi-faceted physician and human being. Certainly there were others who knew Will better than me; however, the shared, and subsequently sequential, experiences with *Critical Care Medicine* provided a common bond. On a personal note, Will suffered from Parkinson’s disease [10], a troublesome medical problem that I have also endured for the last 7 years.

Dr. Shoemaker will be missed. He directly and indirectly touched the lives of many clinicians, scientists, educators, and patients. He was not only appreciated and valued by leaders of the SCCM but also respected by distinguished people in a number of related specialties. It is rare that an individual has the opportunity to initiate, or even be a part of, the genesis of something new. And it’s even more enviable if the new concept evolves into a successful, large, and important entity. Many would point to figures such as Steve Jobs or Bill Gates or other innovators who created disruptive new technologies which changed the world. As an older man now, with Parkinson’s disease, I cannot imagine a more important new innovation than an intensive care unit (ICU). Like Apple and Microsoft changed the world by enabling people to have personal computers and “smart phones”, the ICU envisioned more than 50 years ago by Drs. (Professors) Max Harry Weil, Peter Safar, and William C. Shoemaker and a handful of others [11] has changed the medical world and has become the high quality focal point for the care of critically ill patients. Yes, Dr. Shoemaker will be missed; however, his legacy will live on for many years into the future.

With respect,

Bart Chernow, MD, MACP, Master Fellow ACCP, FCCM,

Editor Emeritus, Critical Care Medicine

Past President, American College of Chest Physicians
